# Immunoprofiles and DNA Methylation of Inflammatory Marker Genes in Ulcerative Colitis-Associated Colorectal Tumorigenesis

**DOI:** 10.3390/biom11101440

**Published:** 2021-09-30

**Authors:** Satu Mäki-Nevala, Sanjeevi Ukwattage, Erkki-Ville Wirta, Maarit Ahtiainen, Ari Ristimäki, Toni T. Seppälä, Anna Lepistö, Jukka-Pekka Mecklin, Päivi Peltomäki

**Affiliations:** 1 Department of Medical and Clinical Genetics, University of Helsinki, FI-00014 Helsinki, Finland; sanjeevi.ukwattage@blueprintgenetics.com (S.U.); paivi.peltomaki@helsinki.fi (P.P.); 2 Department of Gastroenterology and Alimentary Tract Surgery, Tampere University Hospital, FI-33521 Tampere, Finland; erkki-ville.wirta@fimnet.fi; 3 Department of Education and Research, Hospital Nova of Central Finland, FI-40620 Jyväskylä, Finland; maarit.ahtiainen@ksshp.fi (M.A.); jukka-pekka.mecklin@ksshp.fi (J.-P.M.); 4 Department of Pathology, HUSLAB, HUS Diagnostic Center, University of Helsinki and Helsinki University Hospital, FI-00029 Helsinki, Finland; ari.ristimaki@hus.fi; 5 Applied Tumor Genomics Research Program, Research Programs Unit, University of Helsinki, FI-00014 Helsinki, Finland; toni.seppala@helsinki.fi (T.T.S.); anna.lepisto@hus.fi (A.L.); 6 Department of Gastrointestinal Surgery, Helsinki University Hospital, FI-00029 Helsinki, Finland; 7 Department of Sport and Health Sciences, University of Jyväskylä, FI-40014 Jyväskylä, Finland

**Keywords:** Lynch syndrome, ulcerative colitis, colon cancer, immune cell score, DNA methylation, inflammation-associated genes

## Abstract

Immunological and epigenetic changes are interconnected and contribute to tumorigenesis. We determined the immunoprofiles and promoter methylation of inflammation-related genes for colitis-associated colorectal carcinomas (CA-CRC). The results were compared with Lynch syndrome (LS)-associated colorectal tumors, which are characterized by an active immune environment through inherited mismatch repair defects. CA-CRCs (*n* = 31) were immunohistochemically evaluated for immune cell scores (ICSs) and PDCD1 and CD274 expression. Seven inflammation-associated genes (*CD274*, *NTSR1*, *PPARG*, *PTGS2*, *PYCARD*, *SOCS1*, and *SOCS2*), the repair gene *MGMT*, and eight standard marker genes for the CpG Island Methylator Phenotype (CIMP) were investigated for promoter methylation in CA-CRCs, LS tumors (*n* = 29), and paired normal mucosae by multiplex ligation-dependent probe amplification. All but one CA-CRCs were microsatellite-stable and all LS tumors were microsatellite-unstable. Most CA-CRCs had a high ICS (55%) and a positive CD274 expression in immune cells (52%). *NTSR1* revealed frequent tumor-specific hypermethylation in CA-CRC and LS. When compared to LS mucosae, normal mucosae from patients with CA-CRC showed significantly higher methylation of *NTSR1* and most CIMP markers. In conclusion, CA-CRCs share a frequent ICS^high^/CD274^pos^ expression pattern with LS tumors. Elevated methylation in normal mucosa may indicate field cancerization as a feature of CA-CRC-associated tumorigenesis.

## 1. Introduction

Ulcerative colitis (UC) together with Crohn’s disease comprise inflammatory bowel disease (IBD), which is a recognized risk factor for colorectal cancer (CRC) [1]. The development of UC-associated CRC (CA-CRC) is a multifactorial and complex process involving persistent inflammation, alterations in the intestinal microbiome, imbalance of the immune system, and genetic and epigenetic changes [2,3]. Reactive oxygen species and nitrogen intermediates produced by active inflammatory cells promote mutations and genetic instability [4], and cytokine production may promote epigenetic changes contributing to tumorigenesis through DNA repair gene inactivation and other mechanisms [4]. CRC risk in patients with UC is approximately two-fold compared with the average population [3]. Moreover, CA-CRC patients tend to be younger and have multiple tumors, and mucinous and poorly differentiated carcinomas are common [3]. Consistent with the aggressive histopathological characteristics, Watanabe et al. found CA-CRC to be associated with a worse prognosis compared with sporadic colorectal cancer, which might in part reflect diagnosis at late stages [5].

Aggressive histological features, i.e., mucinous and poorly differentiated tumors, also characterize Lynch syndrome (LS)-associated CRC [6], where the cumulative risk of CRC depends on the predisposing mutation: carriers of *MLH1* or *MSH2* germline mutations have over 10-fold relative risks compared to the general population [7]. LS is the most common hereditary condition predisposing to CRC and is caused by pathogenic germline variants in DNA mismatch repair (MMR) genes *MLH1*, *MSH2*, *MSH6*, and *PMS2* [8] or, more rarely, deletions in the 3′ end of the *EPCAM* gene, leading to hypermethylation of the *MSH2* gene promoter [9]. These heterozygous germline defects decrease the levels of functional MMR proteins, compromising their critical cancer avoidance functions and resulting in hypermutated and microsatellite-unstable tumors [10,11]. LS-CRC patients have better survival compared to sporadic CRC [7,12] and tumors are less likely to metastasize, possibly due to an active immune environment of the tumors [6]. A high rate of de novo somatic mutations leads to high levels of neoantigens, causing abundant tumor-infiltrating lymphocytes, which are counterbalanced by the increased expression of immune checkpoint molecules, such as PDCD1 and CD274 [13].

Immunoscore is a method for describing immune responses and is based on CD3+ and CD8+ lymphocyte infiltrations in the tumor center and invasive margin. It has proven to be a strong independent prognostic marker in stage I–III colon cancer [14]. Immune cell score (ICS) was developed following those same principles and produced similar results. High ICS was generally associated with a favorable outcome and differentiated patients with a poor vs. improved prognosis across tumor stages, regardless of MMR status [15,16]. As expected, most MMR-deficient CRCs, whether sporadic or LS, were characterized by high ICS, a profuse amount of PDCD1-positive lymphocytes, and a high expression rate of CD274-positive immune cells [15]. Available information of immune cell infiltration in CA-CRC is limited, and the findings are in part conflicting [17,18,19].

**Figure 1:**
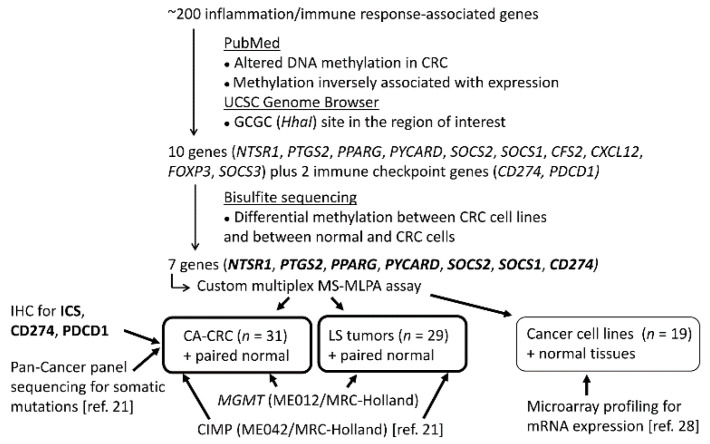
Flow chart of this investigation.

As these observations imply, inflammation has an important role in cancer; however, epigenetic regulation of inflammation-associated genes and its impact on UC- and LS-associated CRC tumorigenesis are not completely understood. Promoter methylation of inflammation-associated genes, such as *CD274*, may be a prognostic factor in sporadic CRC [20]. DNA methylation alterations of inflammation-associated genes could provide a specific mechanism for the widespread inflammatory/immunological alterations that accompany tumorigenesis, especially in association with the CpG island methylator phenotype (CIMP) or microsatellite instability (MSI). In this study, we aimed to evaluate the immunological landscape of CA-CRCs through ICS and altered methylation of inflammation-associated genes, using LS tumors for comparison (Figure 1).

## 2. Materials and Methods

### 2.1. Material

Study material included FFPE specimens of (paired) normal and tumorous colonic mucosa from patients with CA-CRC (normal mucosae, *n* = 17 and carcinomas, *n* = 31) and LS (normal mucosae, *n* = 11, adenomas, *n* = 14, and carcinomas, *n* = 15). Details are presented in Table 1. Sampling was done before any cytostatic cancer treatments, either at the time of the diagnosis or the surgery. All LS adenomas had high-grade dysplasia and their molecular profiles (including MSI, CIMP, and somatic mutational status) were comparable to LS-CRCs, as detailed previously [21]. Therefore, LS adenomas and carcinomas were combined, and the respective results in this study are given for this joint group referred to as “LS tumors”. All LS patients were verified mutation carriers represented in the nationwide Lynch syndrome Registry of Finland. DNA was extracted using the modified extraction protocol of the phenyl–chloroform method [22]. CA-CRC tissue samples were punched and DNA was extracted from these punches (3 × 1 mm), collected from cancerous tissue or histologically normal tissue verified by the pathologist. Normal samples were derived from separate tissue blocks (the whole specimen represented normal tissue by histological evaluation, with one exception, which was punched from a tumor block (adjacent to tumorous tissue)). For LS tissue samples, the pathologist verified the histology and DNA was extracted from the FFPE sections. Normal samples were derived from separate tissue blocks (the whole specimen was histologically evaluated as normal tissue). In tumor samples, the average of tumorous tissue was 46% (SD ± 13.0).

### 2.2. MSI Analysis

MSI status was investigated using mononucleotide repeat markers BAT25 and BAT26, as described in Mäki-Nevala et al. [21]. The diagnosis of MSI required at least one unstable marker.

### 2.3. Immunohistochemical Analysis of PDCD1 and CD274, and Immune Cell Scoring (ICS)

CA-CRC samples were studied for PDCD1 and CD274 expression by immunohistochemical analysis, as described in Ahtiainen et al. [15]. Also, ICS based on CD3 and CD8 expression was calculated according to the previously described protocol [15,16]. Briefly, antibodies used were: anti-PDCD1 (SP269, 1:50; Spring Bioscience, Pleasanton, CA, USA), anti-CD274 (E1L3N, 1:100; Cell Signaling Technology, Danvers, MA, USA), anti-CD3 (LN 10, 1:50; Novocastra, Buffalo Grove, IL, USA), and anti-CD8 (SP16, 1:100; Thermo Fisher Scientific, Waltham, MA, USA). Positively stained cells were analyzed using QuPath [23]. Cut-off values for PDCD1, CD3, and CD8 positivity were determined from a large reference series of CRCs using receiver operating characteristic curves drawn in relation to disease-specific 5-year survival [18]. The cut-off values for PDCD1 were 38 cells/mm^2^ (invasive margin) and 57 cells/mm^2^ (tumor center). Based on these cut-off values, the samples were divided into PDCD1^low^ and PDCD1^high^. Cut-off values for ICS were: 815 for CD3 and 384 for CD8 in the tumor center and 1144 for CD3 and 496 for CD8 in the invasive front. Expressions in the tumor center and invasive front were used to formulate the ICS, as specified in Wirta et al. [16]; one point is given for each section (tumor center or invasive margin with CD3 or CD8 staining) with a lymphocyte count exceeding the determined cut-off value so that in ICS 4, all the sections had a high cell count and in ICS 0, all the sections had a low cell count. Samples were grouped into ICS^low^ (scores 0–2) and ICS^high^ (scores 3–4). Expression of CD274 was evaluated on tumor cells (TC) and tumor-infiltrating immune cells (IC) throughout the tumor center and the invasive margin from stained whole tissue sections. Both the percentage of stained immune and tumor cells and the staining intensity were visually estimated. A sample was considered CD274^pos^ if the proportion of CD274-positive tumor and/or tumor-infiltrating immune cells with moderate or strong intensity exceeded 5%. The location of CD274 expression on tumor and/or immune cells was confirmed by detailed side-by-side analyses of CD274 expression with corresponding HE stained slides.

### 2.4. CpG Island Methylator Phenotype (CIMP) Analysis

Commercial SALSA MS-MLPA ME042-B2 (for LS samples) and ME042-C1 (for CA-CRC samples) probe mixes (MRC Holland, Amsterdam, The Netherlands) were used according to the manufacturer’s protocol. The results were interpreted as described in Mäki-Nevala et al. [21] for CA-CRC and Valo et al. [24] for LS. Briefly, eight established marker genes for CIMP (*CACNA1G*, *CDKN2A*, *CRABP1, IGF2*, *NEUROG1*, *MLH1*, *RUNX3,* and *SOCS1*) were investigated. A set of corresponding normal samples were examined to determine a threshold for hypermethylation for each probe with a stringency level II; these details are described in Valo et al. [24]. CIMP status was assigned by a three-level evaluation [25]. First, probe-level methylation was assessed against the mean Dm of normal mucosae plus two standard deviations (if this value was lower than the technical threshold of 0.15, the technical cut-off was used as a threshold). Second, a gene was considered hypermethylated if at least one-fourth of all probes for that gene were methylated. Third, CIMP(+) status required a minimum of 3/5 marker genes (*CACNA1G*, *IGF2*, *NEUROG1*, *RUNX3*, and *SOCS1*) to be methylated according to the Weisenberger criteria [26].

### 2.5. O-6-Methylguanine-DNA-Methyltransferase (MGMT) Methylation Analysis

The SALSA MLPA Probemix ME012 MGMT-IDH1-IDH2 (MRC Holland, Amsterdam, The Netherlands) was applied according to the manufacturer’s protocol. The probemix included six probes for the *MGMT* promoter area.

### 2.6. Bisulfite Modification and Sequencing

CRC cell lines (HCA7, HCT116, LIM1215, RKO, SW480, and T84) and normal samples (DNA from normal colonic mucosa (Dr. P. Set, Amsbio, Abingdon, United Kingdom) and blood-derived healthy control DNA (Promega, Madison, WI, USA) were bisulfite converted using the EZ DNA Methylation-DirectTM Kit (Zymo Research, Orange, CA, USA) according to the manufacturer’s protocol. Methylation-unbiased primers (Supplementary Figure S1 and Supplementary Table S6) were used to amplify the bisulfite converted DNA samples, and amplified products were sequenced directly or after cloning. NTSR1 was selected for the cloning experiment (Supplementary Figure S2). Amplified DNA samples were cloned into a pCRC2.1 TOPO vector using the TOPO TA Cloning System (Invitrogen, Carlsbad, CA, USA) and One Shot Electrocompetent E. coli (Invitrogen, Carlsbad, CA, USA). Resulting white colonies were used for the purification of DNA and were sequenced, totaling 14–30 sequences per amplification product. The frequency of methylated sites was calculated and compared to MS-MLPA results (Supplementary Figure S2).

### 2.7. Custom-Made Methylation Specific Multiplex Ligation-Dependent Probe Amplification (MS-MLPA) Assay for Inflammation-Related Genes

The inflammation-associated gene panel was designed in-house (Figure 1). We started with a list of more than 200 inflammation and/or immune response-associated genes in mice (Paloviita, 2015, personal communication and MSc thesis, 2016). An extensive literature review was conducted in the PubMed using the following search terms: “gene_name” AND “methylation” AND “cancer”. in October and November 2016. Human orthologs of those inflammation-related genes whose DNA methylation alterations were associated with CRC and inversely associated with mRNA and/or protein expression were characterized. For a gene to be considered further, it was necessary that at least one GCGC site occurred in the region of interest to allow the subsequent design of an MS-MLPA probe. We selected the most interesting genes (*n* = 10); additionally, the immune checkpoint genes *CD274* and *PDCD1* were included, although to that date, there were no studies reporting methylation alterations associated with CRC. For these 12 genes, bisulfite sequencing primers were designed or previously published ones were used for the regions (Supplementary Table S6). CpG sites and promoter regions were identified using the UCSC Genome Browser [27]. Bisulfite sequencing was performed as described above. MS-MLPA probes were designed if distinct methylation profiles were observed between various CRC cell lines and in normal controls compared to CRC cell lines.

The final seven genes were: *CD274* (Cluster of Differentiation 274), also known as the gene for Programmed Cell Death Ligand-1 (*PD-L1*); *NTSR1* (Neurotensin Receptor 1); *PPARG* (Peroxisome Proliferator-Activated Receptor Gamma); *PTGS2* (Prostaglandin-Endoperoxide Synthase 2), also known as cyclo-oxygenase 2 (*COX-2*); *PYCARD* (PYD and CARD Domain Containing Protein), *SOCS1* (Suppressor of Cytokine Signaling 1), and *SOCS2* (Suppressor of Cytokine Signaling 2). MS-MLPA probes were designed according to the protocol by the MRC-Holland (Amsterdam, The Netherlands) (version 15) (Supplementary Figure S1 and Supplementary Table S1). Probes were ordered from the Integrated DNA Technologies (Coralville, IA, USA), and were tested and optimized against bisulfite sequencing results. The optimized custom-made probe mix including probes for the aforementioned seven genes was used together with the SALSA MLPA probe mix P300-B1 Reference2 (MRC-Holland, Amsterdam, The Netherlands) and the SALSA MLPA EK-FAM reagent kit (MRC-Holland, Amsterdam, The Netherlands) according to the manufacturer’s protocol. Cut-off values for hypermethylation were calculated in the same way as described for the CIMP panel [24]. To note, *SOCS1* is also included in the commercial CIMP panel (see above), but its targeted region is different; CIMP panel probes are located 1374-2441bp downstream from the custom-made probe.

### 2.8. mRNA Expression Analysis

Cancer cell lines’ mRNA expression was assessed with Affymetrix Human Genome U133 Plus 2.0 GeneChip^®^ microarrays (Affymetrix, Santa Clara, CA, USA), as described [28]. Normal tissue RNA was purchased from Amsbio (Abingdon, UK). Microarray data were analyzed by the Affy package in R using RMA (robust multi-array average) normalization methods.

### 2.9. Statistical Analysis

Statistical analyses were conducted using the SPSS software, version 25.0 and 27.0 (IBM SPSS Inc., Chicago, IL, USA). Fisher’s exact test was used to study pairwise comparisons of categorical variables. Normal distribution of continuous data was tested using the Shapiro–Wilk test. Continuous variables were analyzed using the independent *t*-test for normally distributed variables and the non-parametric Mann–Whitney–U test was applied on not normally distributed variables. Similarly, correlation analyses were calculated either with Pearson’s or Spearman’s correlation test. Exact two-sided *p* values were calculated. When applicable, raw *p* values were corrected with the Bonferroni method for multiple comparisons. Corrected *p* values < 0.05 were considered statistically significant and are presented throughout the paper, unless otherwise indicated.

## 3. Results

### 3.1. Immunoprofiles of CA-CRC

**Figure 2:**
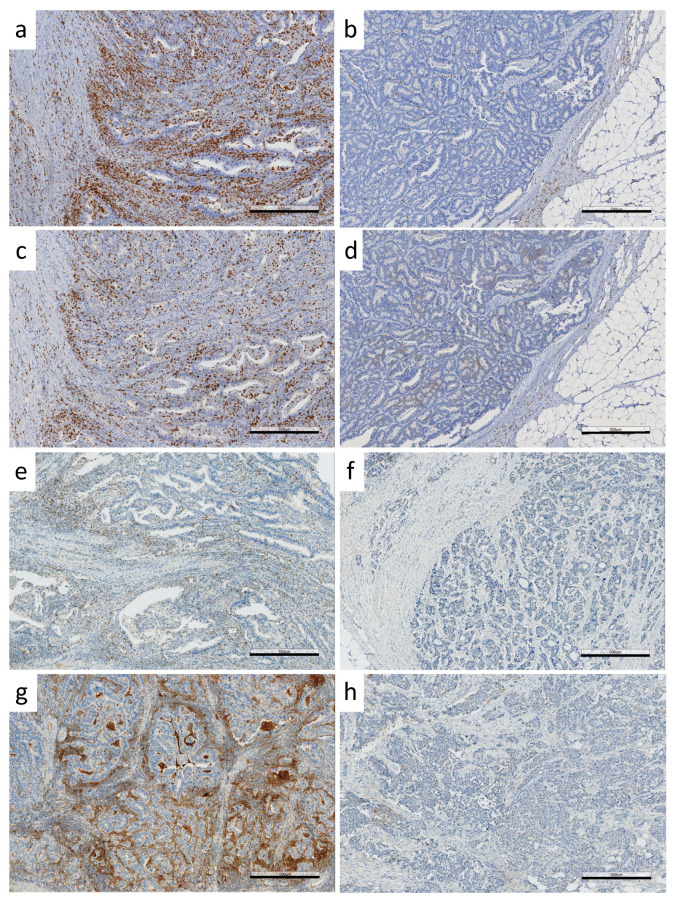
IHC staining of immunoproteins. Examples of (**a**) CD3 high, (**b**) CD3 low, (**c**) CD8 high, (**d**) CD8 low, (**e**) PDCD1 high, (**f**) PDCD1 low, (**g**) CD274 (IC) high and (**h**) CD274 (IC) low expressions. Black bars in the right bottom corners indicate the size of 500 µm.

Immunoprofiles were determined based on ICS and immune checkpoint protein expression. This study comprised 31 CA-CRCs, all microsatellite-stable (MSS) except for one (Table 1). ICSs were distributed as follows: 0 in 23%, 1 in 10%, 2 in 13%, 3 in 16%, and 4 in 38% of the tumors. On a dichotomous scale, ICS was high (three or four) in 17/31 (55%) CA-CRC-tumors (Table 2). Figure 2 represents high and low expressions of CD3, CD8, PDCD1, and CD274 by IHC.

Most CA-CRCs showed a high expression of the immune checkpoint protein PDCD1: 18/31 (58%) when PDCD1-positive lymphocytes in the tumor center were counted (Table 2) and 21/31 (68%) based on PDCD1-positive lymphocytes in the invasive margin. Half of the CA-CRCs (16/31, 52%) expressed CD274-positive immune cells, whereas tumor cells were CD274-negative.

We combined the data of tumor-infiltrating lymphocytes, i.e., ICSs, and the CD274 expression of immune cells, as suggested and done in previous studies [15,29]. The samples were divided into four subgroups: ICS^high^/CD274^pos^ (type I; adaptive immune resistance), ICS^low^/CD274^neg^ (type II; immunological ignorance), ICS^high^/CD274^neg^ (type III; tolerance), and ICS^low^/CD274^pos^ (type IV; intrinsic induction) according to Teng et al. [29]. Details are presented in Table 2.

In this investigation, ICS, PDCD1, and CD274 assays were performed on CA-CRC alone. We previously [15] immunologically profiled a separate set of LS-CRCs (all from verified carriers of pathogenic germline mutations of *MLH1*, *MSH2*, or *MSH6*), together with cohorts of sporadic CRCs, by the same method used here, enabling direct comparisons (Table 2). CA-CRC did not significantly differ from LS-CRC relative to any immunological parameter assessed. As 97% of CA-CRCs were MSS, we selected the MMR-proficient (pMMR) subgroup of sporadic CRCs for comparison. In CA-CRCs, CD274 on immune cells was more often positive and ICS high (the difference reached statistical significance for CD274), resulting in a significantly different distribution of ICS/CD274^IC^ types between CA-CRC and pMMR-CRC (Table 2).

### 3.2. Immunoprofile vs. Somatic Mutational Status and CIMP

**Figure 3:**
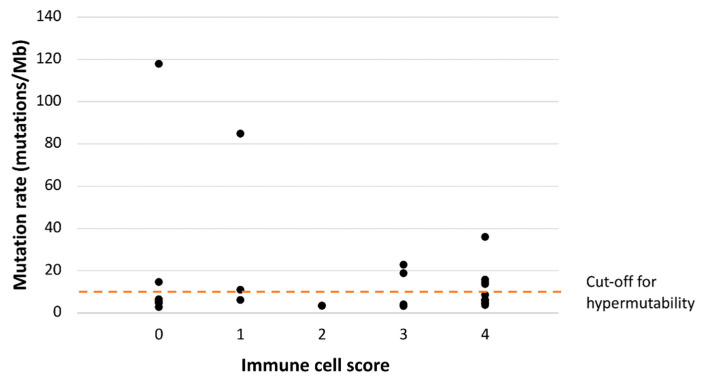
Somatic mutation rates plotted against ICS in CA-CRC. Somatic mutations consisted of somatic non-synonymous mutations with VarScan2 (Genome Institute, Washington University, St. Louis, MO, USA) *p* value < 0.05 (27 tumors sequenced in our previous study were included in this analysis).

Of the 31 CA-CRCs, 27 were sequenced for somatic mutations as part of our previous study [21], which allowed us to compare mutational status and immunoprofiles. CA-CRC tumors broke down into three subgroups based on their status of MSI and hypermutability (the latter defined as over 10 nonsynonymous somatic mutations/Mb): group one comprised hypermutated MSI tumors (*n* = 1); group two, hypermutated MSS tumors (*n* = 9); and group three, non-hypermutated MSS tumors (*n* = 17). We plotted the number of somatic mutations against ICS, and all ICS groups, except number two, included two or three hypermutated tumors (Figure 3). Interestingly, two tumors (including the single one with MSI) had somatic mutation numbers around the “ultramutated” range (100 mutations/Mb) and both had low ICS values (0 and 1). The mutation rate (mutations/Mb) was not associated with the immunological subgroup defined by ICS and CD274 expression (*p* = 0.835). The ten hypermutant tumors were distributed among all four immunological subgroups, most commonly ICS^high^/CD274^pos^ (four tumors) and ICS^low^/CD274^neg^ (three tumors, including the single MSI tumor), followed by ICS^high^/CD274^neg^ (two tumors), and ICS^low^/CD274^pos^ (one tumor).

Of all CA-CRC tumors, 12 (39%) were CIMP(+) and 19 were CIMP(−) (61%) (Table 1). CIMP status was not associated with the immunological subgroup (*p* = 0.486). The largest proportion of CIMP(+) tumors (6/12, 50%) fell into the ICS^high^/CD274^pos^ group and of CIMP(−) tumors (6/19, 32%), into the ICS^low^/CD274^neg^ group.

### 3.3. Methylation of Inflammation-Related Genes in CA-CRC and LS Tumors

We focused on seven inflammation-associated genes selected, as described in Figure 1 and Methods (Section 2.7). Our custom-made MS-MLPA panel (Supplementary Figure S1 and Supplementary Table S1) was validated against bisulfite sequencing results of cell lines. A cloning experiment was performed for one of the genes, *NTSR1*. Quantification of methylation by MS-MLPA vs. cloning yielded closely concordant results (Supplementary Figure S2).

**Figure 4:**
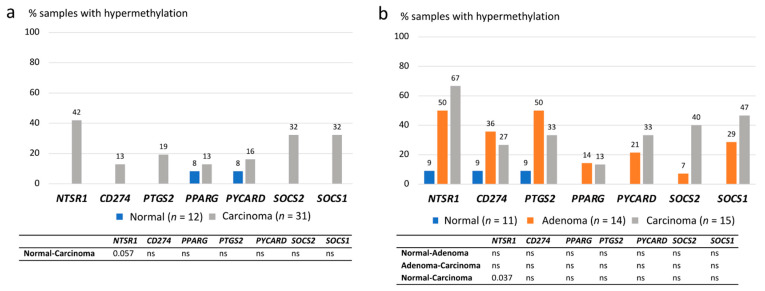
Hypermethylation frequencies of inflammation-associated genes. (**a**) Hypermethylation frequencies in CA-CRC samples. (**b**) Hypermethylation frequencies in LS-associated samples. Bonferroni-corrected two-sided *p* values are presented in the tables below for pairwise comparisons, and ns stands for non-significant *p* value. Missing frequency values of the normal sample group indicate the hypermethylation frequency of 0%.

Methylation data were evaluated in two ways: first, by determining the frequencies of hypermethylation in tumor tissues relative to gene-specific thresholds derived from normal mucosa (Supplementary Table S2), and second, by using the degrees of methylation (based on methylation dosage ratios (Dm) obtained for each gene in each specimen) as continuous variables. In the former analysis, both CA-CRC and LS-associated colorectal samples showed hypermethylation of tumor samples compared with normal mucosae (Figure 4a,b). In CA-CRC, the hypermethylation frequencies of *NTSR1*, *SOCS2,* and *SOCS1* (32–42%) clearly exceeded those of the paired normal tissues (0%); however, statistical significances were lost when the Bonferroni correction was applied (Figure 4a), and only *NTSR1* remained borderline significant (*p* = 0.057). Among LS samples, *NTSR1*, *SOCS2*, and *SOCS1* showed tentatively increased methylation frequencies in carcinomas compared to normal mucosae, but only *NTSR1* remained significant after adjustment for multiple testing (*p* = 0.037) (Figure 4b). *NTSR1* and *PTGS2* hypermethylation were elevated already in adenomas and the latter one showed an even somewhat higher hypermethylation frequency in adenomas compared to carcinomas, 50% vs. 33% (Figure 4b).

**Figure 5:**
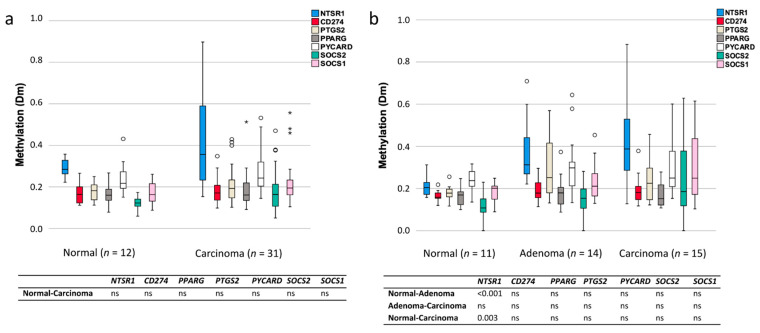
Comparison of the degrees of methylation of inflammation-associated genes in normal colon and tumor specimens. (**a**) Distribution of methylation dosage (Dm) values in CA-CRC samples. (**b**) Distribution of methylation dosage values in LS samples. The upper and lower edges of the boxes are the 75th and 25th percentiles, the horizontal line inside the box indicates the median, the whiskers denote the highest and lowest values, and the asterisks and open circles represent outliers: extreme values and potential outliers, respectively (based on the interquartile range (IQR)). Bonferroni-corrected two-sided *p* values are presented in the tables below for pairwise comparisons, and ns stands for non-significant *p* value.

Treating the degrees of methylation as continuous variables, *NTSR1* and *SOCS2* revealed the most evidently increased methylation levels in CA-CRCs compared to normal mucosa, although no statistical significance was reached (Figure 5a). *NTSR1* methylation values were characterized by a high variance, which suggested the existence of two subsets of tumor samples, one with increased and another one with decreased methylation compared to normal colon samples. In LS, *NTSR1* had significantly increased methylation values in tumor samples compared to normal counterparts, both in adenomas (*p* < 0.001) and carcinomas (*p* = 0.003) (Figure 5b).

When the hypermethylation frequencies of inflammation-associated genes were stratified by CIMP status, higher (although statistically non-significant) hypermethylation frequencies were seen for the CIMP(+) vs. CIMP(−) subsets of CA-CRCs for *PYCARD* (33 vs. 5%), *CD274* (25 vs. 5%), *NTSR1* (50 vs. 37%), and *SOCS2* (42 vs. 26%). The degree of methylation as a continuous variable was not significantly associated with the CIMP status for any inflammation-associated gene in CA-CRC. Among LS tumors, *NTSR1* methylation was associated with overall CIMP: the average degree of methylation was 0.62 in CIMP(+) tumors vs. 0.34 in CIMP(−) tumors (*p* = 0.002).

Intriguingly, normal mucosa samples of CA-CRC vs. LS patients differed significantly: the average degree of methylation for *NTSR1* was significantly higher in normal mucosa from CA-CRC patients (mean Dm ± SD of 0.29 ± 0.12, range 0.22–0.36) compared to normal mucosa from LS patients (0.21 ± 0.15, range 0.16–0.31) (*p* = 0.007). In the corresponding tumors, the average degrees of methylation of *NTSR1* or other inflammation-associated genes did not significantly differ between UC and LS patients. When compared to normal mucosae of LS patients, normal mucosae of CA-CRC patients additionally showed significantly increased average degrees of methylation in at least one of the probes for all CIMP marker genes except *MLH1* (Supplementary Table S3). A similar trend was seen for the corresponding tumor tissues.

Methylation of inflammatory marker genes was not significantly associated with immunological subgroup or ICS. None of the inflammatory marker probes showed an age-related correlation with methylation levels in CA-CRC tumors, and the same was true for CIMP markers (in LS tumors, one CIMP probe, *IGF2* I, showed statistical significance (*r* = 0.612, *p* = 0.013). Similarly, no age-related correlation with methylation was observed in normal mucosae (*n* = 12) from CA-CRC patients (in LS samples (*n* = 11), *RUNX3* I and *RUNX3* II showed a significant, but inverse correlation between age and methylation (*r* = −0.858, *p* = 0.022, and *r* = −0.841, *p* = 0.037, respectively)). The tumor stage was not associated with the hypermethylation frequency or methylation levels of inflammation-associated genes or CIMP markers in CA-CRC or LS tumors.

### 3.4. Methylation of MGMT

**Figure 6:**
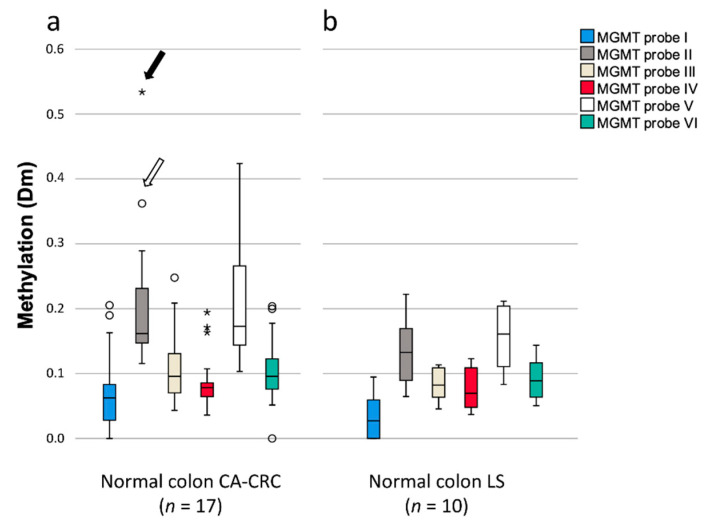
*MGMT* methylation in normal mucosa specimens. (**a**) Methylation degrees in normal colon of CA-CRC patients. (**b**) Methylation degrees in normal colon and LS patients. Arrows in Figure 6a denote cases discussed in the text, with higher Dm values in normal than tumor tissues. The upper and lower edges of the boxes are the 75th and 25th percentiles, the horizontal line inside the box indicates the median, the whiskers denote the highest and lowest values, and the asterisks and open circles represent outliers: extreme values and potential outliers, respectively (based on the interquartile range (IQR)).

Prompted by the observations of elevated methylation of *NTSR1* and CIMP marker genes as a feature of normal mucosae from CA-CRC patients (Figure 5, Supplementary Table S3), the repair gene O-6-Methylguanine-DNA Methyltransferase (*MGMT*) was investigated for promoter methylation, as it has been proposed to serve as an early marker for colorectal cancer [30]. In this investigation, probes III (263 bp before exon 1) and V (73 bp after exon 1) represent methylation hot spots closely associated with silencing of the *MGMT* gene [31]. No significant difference was seen between normal mucosae and tumors from either CA-CRC or LS patients (Supplementary Table S4), and CIMP status did not associate with the average degree of methylation of any *MGMT* probes. Figure 6 shows the distributions of the degrees of methylation for the different *MGMT* probes for normal mucosae specifically. In comparison with LS, most of the probes had higher methylation levels in CA-CRC patients, especially *MGMT* II, although statistical significance was not reached (*p* = 0.100). Some CA-CRC patients’ normal samples showed very high levels of methylation, exceeding the levels seen in their matched tumors; for probe II, for example, one case showed a Dm of 0.53 in normal tissue (solid arrow in Figure 6a) vs. 0.27 in tumor tissue, and in another case, a Dm of 0.36 in normal tissue (open arrow in Figure 6a) vs. 0.28 in tumor tissue. A similar phenomenon was not seen in LS (Figure 6b). These methylation alterations could serve as field defects in CA-CRC-related tumorigenesis.

### 3.5. Methylation of Inflammation-Related Genes in Cancer Cell Lines and Association with Gene Expression

To evaluate the functional impact of CpG sites studied in inflammatory marker genes, cancer cell line-derived and normal reference DNA samples were investigated for methylation by custom-made MS-MLPA (Supplementary Table S5), and the data were combined with gene expression microarray data (Supplementary Figure S3). The ovarian cell lines ES2, CAOV3, and SKOV3, the uterine cancer cell line of AN3CA, and the colorectal cancer cell lines HCT15, RKO, HCA7, LIM1215, SW48, LoVo, and KM12 were included. When mRNA expression values (log2 fold change) were plotted against the methylation dosage values (Dm), a negative correlation was observed for all probes. A statistically significant correlation between mRNA expression and methylation was found for *CD274* (*r* = −0.680, *p* = 0.010), *NTSR1* (*r* = −0.680, *p* = 0.010), *PTGS2* (*r* = −0.735, *p* = 0.004), and *PYCARD* (*r* = −0.564, *p* = 0.045) (Supplementary Figure S3). The data indicate that DNA methylation of genes and regions studied is likely to regulate gene expression.

## 4. Discussion

Many key cytokines and transcription factors required for the differentiation and function of central component cells of innate and adaptive immunity are epigenetically regulated; conversely, altered immune responses and inflammation can drive epigenetic reprogramming [32,33]. We set out to investigate the relationship between immunological landscapes and altered DNA methylation in colorectal tumors arising in the background of inflammation (CA-CRC) or immunological activation (LS). We observed similarities and differences that suggest in part shared and in part distinctive roles for immunological and epigenetic factors in these two disease entities.

Quantification of CD3+ and CD8+ lymphocytes in the tumor core and invasive margins has turned out useful in predicting a prognosis and the response to immunotherapy [34]. Previous immunoprofiling studies on IBD-associated CRCs arrived at conflicting results. Michael-Robinson et al. [17] observed significantly higher CD3+ and CD8+ tumor-infiltrating lymphocyte counts in IBD (UC and Crohn’s disease)-associated neoplasia compared to sporadic CRC; both groups consisted of MSS tumors. In contrast, Soh et al. [19] found that IBD-associated CRCs had significantly lower levels of cells expressing CD3, CD8, and CD274 in immune cells compared to sporadic CRC (both groups included ~15% fractions of MSI cases). The choice of antibodies, diverse cut-off values used for a positive result, and the clinicopathological features of tumors may in part explain the observed variation in results [34]. We tested several commercial antibodies for immunoprofiling, and the final selection was based on performance [15]. The ICS values of our CA-CRCs (97% of which were MSS) did not significantly differ when compared to MSS sporadic CRCs or MSI LS-CRCs from our previous investigation [15] (Table 2). However, the distributions of ICS/CD274^IC^ categories were significantly different between CA-CRC and pMMR sporadic CRC: in CA-CRC, ICS^high^/CD274^pos^ was the most frequent category (35%), as opposed to ICS^low^/CD274^neg^ (52%) in sporadic CRC. In spite of contrasting MMR statuses, the immunological pattern of CA-CRCs resembled that of LS-CRCs to a considerable extent and no significant differences between CA-CRC and LS-CRC were seen (Table 2). This investigation was not designed to study the cellular subtype specificity of CD274 expression on immune cells. With the accuracy of IHC, we can assume that the CD274-positive immune cells in CA-CRCs were mainly macrophages, as shown previously by multilabel immunofluorescent analysis of selected CRC samples [15].

A high somatic mutational burden characteristic of MMR-deficient CRCs and *POLE*-mutant MMR-proficient (pMMR) CRCs has been found to correlate with a high neoantigen load and high levels of CD8+ and CD4+ T-cells, predicting responsiveness to immune checkpoint inhibitors [35]. The relationship between mutational load and ICS in our CA-CRCs did not comply with this basic rule (Figure 3). Our CA-CRCs included a hypermutant (*n* = 10, 37%) and a non-hypermutant subset (*n* = 17, 63%) [21]. The frequency of ICS^high^ tumors was only slightly higher in the hypermutant subset (6/10, 60%, vs. 9/17, 53%). Moreover, two tumors had especially high mutation numbers (118 and 85/Mb); both fell into the ICS^low^ (ICS 0–2) category (Figure 3) and were late-stage tumors (III or IV). Not all cancer types show a positive correlation between CD8 T-cell levels and tumor mutational burden, and such cancers may not respond to an immune checkpoint blockade [36].

The coordinated methylation of CpG island promoters (CIMP) represents an epigenetic mechanism able to affect many CRC-relevant genes simultaneously, including those involved in immune responses and inflammation. CIMP frequencies depend on the method and classification system used. We adopted our CIMP scoring method from Berg et al. [25] which resulted in the CIMP(+) frequency of 39% for our CA-CRCs (this frequency is within the range obtained for sporadic CRCs by Berg et al. [25]). CIMP status was not significantly associated with immunological subgroup; however, our observation of the largest proportion of CIMP(+) vs. CIMP(−) tumors falling into distinct immunological subgroups (the former into ICS^high^/CD274^pos^ vs. the latter into ICS^low^/CD274^neg^) suggests that a possible modulating role of CIMP on immunological patterns cannot be totally excluded.

For *CD274*, we had methylation data on patient samples (CA-CRC and LS) and cell lines, mRNA expression data on cell lines, and protein expression data on CA-CRC tumors. In tumors, increased [37] and decreased [38] methylation relative to the corresponding normal tissues has been noted, but the difference may not always be marked [38,39] in agreement with our observations (Figure 4 and Figure 5). *CD274* promoter methylation is inversely correlated with mRNA expression [20,37], in compliance with our cell line findings (Supplementary Figure S3). While *CD274* mRNA and protein expression may not accurately correlate with each other [37], observations of promoter hypomethylation being associated with CD274 overexpression in cancer are especially interesting from the scientific and clinical points of view. Franzen et al. [38] dichotomized *CD274* methylation levels in tumors according to the median, which resulted in *CD274*-low and *CD274*-high groups; CD274 protein positivity was significantly associated with the former group. In our CA-CRC tumors, no such association was found, which was not surprising considering the narrow distribution of *CD274* methylation degrees around the median (Figure 5a).

Of inflammation-associated genes, *NTSR1* showed the most evident alterations between normal mucosae and tumors in both CA-CRC and LS patients (Figure 4 and Figure 5). *NTSR1* signaling has been linked to tumorigenesis in various cancers, such as lung, breast, and colon [40,41]. The signaling is involved in cancer cell proliferation, survival, migration, invasion, and metastasis [42]. *NTSR1* acts like an oncogene in many cancers, but its hypermethylation in colon tumors suggests a role as a tumor suppressor as well [40]. Hypermethylation of *NTSR1* in CRC was associated with less invasive growth and better overall survival in the study of Kamimae et al. [40]. The wide distribution of degrees of methylation (Dm values) in our CA-CRC tumors (Figure 5) suggested the presence of two subgroups: one with *NTSR1* hypermethylation and one with hypomethylation relative to normal mucosa. Our CA-CRCs included 13 hypermethylated tumors defined as having *NTSR1* Dm values equal to or higher than the average Dm in reference normal mucosae plus two standard deviations (Figure 5a). Four (31%) of the hypermethylated tumors were diagnosed at advanced stages (III or IV). Tumors with Dm equal to or below the average Dm of normal mucosae minus two standard deviations were considered hypomethylated. Among a total of four such CA-CRC tumors, two (50%) were diagnosed at advanced stages. Thus, the observed trend was in line with the suggestion of a more favorable outcome for hypermethylated than hypomethylated cases [40] and warrants additional investigations with larger sample sizes.

Molecular alterations that develop in histologically normal mucosa and have the potential to progress to dysplasia and cancer are referred to as “field defects”. Such defects are a well-recognized phenomenon in UC. Inactivating somatic mutations in genes that normally downregulate pro-inflammatory signals were recently described in the inflamed intestinal mucosa from UC patients [43]. Chronic inflammation can also epigenetically alter epithelial cells; both hypomethylated [44] and hypermethylated [45] fields have been described. Compared to genetic fields, the clonality of epigenetic field defects is difficult to assess given the reversible nature of epigenetic alterations. In our investigation, methylation levels of *NTSR1* (Figure 5) and at least one of the probes for all CIMP marker genes except *MLH1* (Supplementary Table S3) were significantly higher in CA-CRC-derived normal mucosae compared to LS-derived normal mucosae, suggesting the existence of hypermethylated fields in the normal mucosae from CA-CRC patients. Age-related methylation may cause field defects, but there was no age difference between our CA-CRC and LS patients (Table 1). Our CA-CRC patients showed a significantly higher average degree of *IGF2* methylation in normal mucosa when compared to LS patients (Supplementary Table S3). *IGF2* is one of the genes whose promoter methylation increases with aging, causing predisposition to colorectal neoplasia [46]. In our study, too, methylation levels of this gene (probe *IGF2* I) increased along with age in both CA-CRC (*r* = 0.225) and LS tumors (*r* = 0.612), but significantly only in LS tumors. Our normal tissues originated from tissue blocks separate from tumors blocks (see Section 2.1), except for one that was derived from the immediate vicinity of tumorous tissue. We investigated this sample for methylation (inflammation-associated genes, CIMP, and *MGMT*), and this sample showed methylation degrees comparable to average normal samples from CA-CRC patients.

Available results concerning the methylation of the repair gene *MGMT* in UC-related tumorigenesis are inconsistent. Svrcek et al. [47] detected a loss of *MGMT* protein expression in colonic mucosa from over 70% of IBD patients (with or without CRC), which significantly exceeded the frequencies observed in CRC patients without IBD. *MGMT* promoter methylation by methylation-specific PCR (a qualitative method) showed an inverse correlation with expression, although apparently discrepant cases (loss of expression without methylation and expressed protein despite promoter methylation) were also seen, especially in CA-CRC patients [47]. Svrcek et al. [47] hypothesized that an *MGMT* field defect may trigger CRC through G:C to A:T mismatches targeting *KRAS*, or through *MLH1* methylation and deficient MMR. On the other hand, studies exist in which no difference in *MGMT* methylation between UC-related and non-inflammatory colon tumorigenesis pathways was observed [48,49]. In our investigation, *MGMT* methylation did not significantly differ between normal mucosa and tumor tissues, arguing against a major role of *MGMT* in UC-related colon tumorigenesis. Possible dilution with normal cells was unlikely to explain the lack of hypermethylation in tumor cells since we observed no correlation when tumor cell percentage was plotted against methylation degrees of MGMT probes. A few CA-CRC patients showed very high methylation levels in normal mucosa (Figure 6a), possibly serving as field defects in those patients. Interestingly, the case marked with a solid arrow in Figure 6a had a somatic *KRAS* G>A substitution (Gly12Asp mutation, ref. [21]) in tumor tissue, supporting the hypothesis of Svrcek et al. [47].

Our CA-CRC and LS samples were formalin-fixed, paraffin-embedded (FFPE), and the chosen approaches were tailored to archival samples. Limitations of this study include relatively small sample sizes and a reliance on targeted methylation data. Additionally, the correlation between methylation and expression could be addressed by cell line studies alone instead of patient specimens. Furthermore, many of our CA-CRC patients had received immunosuppressive medication, and stratification by treatment history was not possible. Our findings need to be confirmed in larger cohorts of CA-CRC patients, preferably taking treatment histories into account.

In conclusion, our study revealed, first, ICS^high^/CD274^pos^ as the most frequent immunological subcategory in CA-CRCs, thereby resembling LS-CRCs, and second, frequent epigenetic field defects in non-neoplastic mucosa of CA-CRC patients. From the clinical point of view, the immunological subtype may be relevant for immunotherapy [35]. Epigenetic field defects may provide early biomarkers of carcinogenesis and identify UC patients who might benefit from more intensive surveillance [50].

## Figures and Tables

**Table 1:** Characteristics of the patient samples.

Characteristics	CA-CRC	LS Tumors Combined ^1^	LS Adenomas (High-Grade Dysplasia)	LS Carcinomas	*p* Value CA-CRC vs. LS Tumors Combined ^1^
No. of patients	31	24	14	12	NA
Male sex ^2^	20 (64%)	9 (38%)	5 (40%)	5 (42%)	0.060
Age, years (mean ± SD) ^2^	51.4 (±10.7)	51.2 (±14.1)	49.2 (±15.2)	53.6 (±12.3)	0.948
Years of colitis before CRC diagnosis (mean ± SD) ^2^	23.8 (±10.8) ^3^	-	-	-	NA
Gene mutated in germline ^2^					
*MLH1*	-	18 (75%)	12 (86%)	8 (67%)	NA
*MSH2*	-	3 (13%)	1 (7%)	2 (17%)	NA
*MSH6*	-	3 (13%)	1 (7%)	2 (17%)	NA
No. of tumors	31	29	14	15	NA
MSI tumors	1 (3%)	29 (100%) ^4^	14 (100%) ^4^	15 (100%) ^4^	<0.00001
CIMP(+) tumors	12 (39%)	7 (24%)	2 (14%)	5 (33%)	0.274
Tumor location					
Proximal ^5^	13 (42%)	17 (59%)	6 (43%)	11 (73%)	0.431
Distal	15 (48%)	12 (41%)	8 (57%)	4 (27%)	
NA	3 (10%)	0	0	0	
Stage of carcinomas					
I	13 (42%)	10 (67%)	-	10 (67%)	0.190
II	6 (19%)	3 (20%)	-	3 (20%)	
III	9 (29%)	1 (7%)	-	1 (7%)	
IV	3 (10%)	0	-	0	
NA	0	1 (7%)	-	1 (7%)	

^1^: LS-associated adenomas and carcinomas combined. ^2^: Calculated per patients. ^3^: Information for eight cases not available. ^4^: MSI information for one adenoma and three carcinoma cases not available, but immunohistochemical analysis suggested MMR defect in the samples. ^5^: From caecum to splenic flexure (included). Note regarding LS samples: Multiple samples were available from three LS patients (three carcinomas and two carcinomas from one patient each, and a carcinoma plus adenoma from two patients each). If sampling took place at different time points (metachronous neoplasias), different ages were included in the calculation of age at diagnosis. Abbreviations: CA-CRC, colitis-associated colorectal cancer; CIMP, CpG island methylator phenotype; CRC, colorectal cancer; LS, Lynch syndrome; MSI, microsatellite unstable; NA, not available or applicable; SD, standard deviation.

**Table 2:** PDCD1, CD274, and ICSs in CA-CRC (this study), compared with sporadic pMMR-CRC and LS-CRC from our previous investigation.

		Ahtiainen et al. [15]		*p* Value	
Immunoprofile Characteristics	CA-CRC	pMMR-CRC	LS-CRC	CA-CRC vs.	
	(*n* = 31)	(*n* = 100)	(*n* = 48)	pMMR-CRC	LS-CRC
PDCD1 ^1^					
Low	13 (42%)	51	17 (35%)	ns	ns
High	18 (58%)	49	31 (65%)		
CD274 on tumor cells					
Negative	31 (100%)	99	45 (94%)	ns	ns
Positive	0	1	3 (6%)		
CD274 on immune cells					
Negative	15 (48%)	70	18 (37%)	0.033	ns
Positive	16 (52%)	30	30 (63%)		
ICS					
Low (0–2)	14 (45%) ^2^	60	13 (27%)	ns	ns
High (3–4)	17 (55%) ^3^	40	35 (73%)		
ICS/CD274^IC^ (immunological group) ^4^					
ICS^high^/CD274^pos^ (type I)	11 (35%)	16	21 (44%)	0.023	ns
ICS^low^/CD274^neg^ (type II)	9 (29%)	52	9 (19%)		
ICS^high^/CD274^neg^ (type III)	6 (19%)	25	14 (29%)		
ICS^low^/CD274^pos^ (type IV)	5 (16%)	7	4 (8%)		

^1^: PDCD1 classification was based on PDCD1-positive lymphocytes in tumor center (cut-off 57 cells/mm^2^ for CA-CRC and 55 cells/mm^2^ for the remaining groups), ^2^: ICS was 0 for 7 (23%), 1 for 3 (10%), and 2 for 4 (13%) CA-CRCs. ^3^: ICS was 3 for 5 (16%) and 4 for 12 (38%) CA-CRCs. ^4^: CD274 expression in tumor-infiltrating immune cells. Abbreviations: IC, immune cell; pMMR, MMR-proficient; ns, non-significant.

## Data Availability

The mRNA expression profiling data is publicly available at the GEO (accession number: GSE58058). The original methylation datasets used and/or analyzed during the current study are available from the corresponding author on reasonable request.

## Supplementary Materials

The following are available online at https://www.mdpi.com/article/10.3390/biom11101440/s1, Figure S1: Illustration of CpG islands and investigated genomic regions of inflammation-associated genes included in the MS-MLPA panel, Figure S2: *NTSR1* cloning experiment compared to MS-MLPA, Figure S3: Correlation between expression and methylation of inflammation-associated genes in cell lines, Table S1: MS-MLPA probes, Table S2: Cut-off methylation dosage ratios for hypermethylation, Table S3: Average methylation dosage ratios and their standard deviations of each CIMP markers, Table S4: Average methylation dosage ratios and standard deviations of each MGMT probe, Table S5: Methylation dosage ratios of inflammation-associated genes in cancer cell lines, Table S6: Bisulfite sequencing primers.
