# Supplementary material for: Immunoprofiles and DNA Methylation of Inflammatory Marker Genes in Ulcerative Colitis-Associated Colorectal Tumorigenesis
Source: Biomolecules. 2021 Sep 30;11(10):1440. doi: 10.3390/biom11101440 (PMC8533626; doi:10.3390/biom11101440)
Supplement: Supplementary file 1 [file biomolecules-11-01440-s001.zip › biomolecules-1367274-supplementary.pdf]

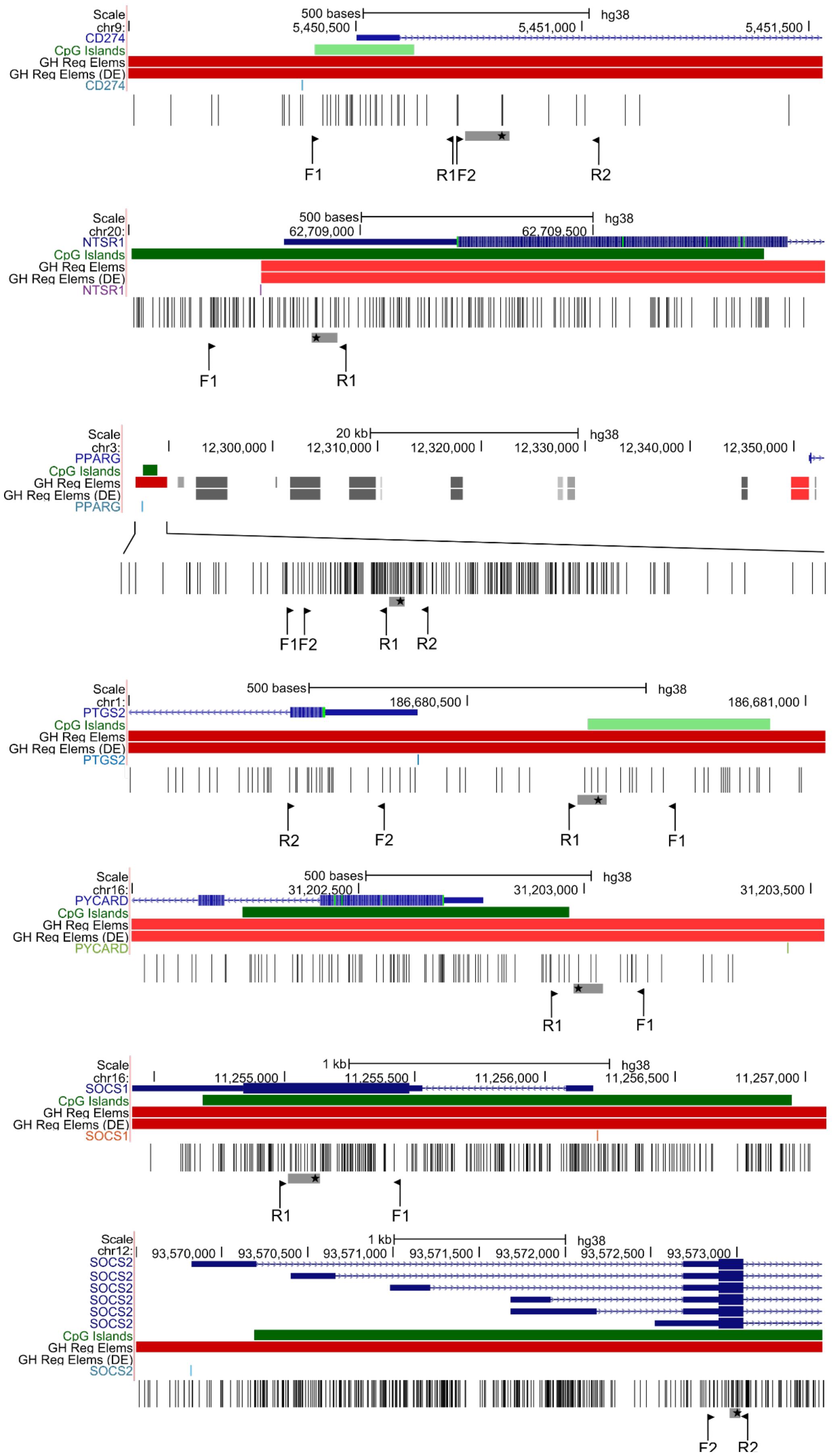

Supplementary Figure S1. Illustration of CpG islands and investigated genomic regions of inflammation-associated genes included in the MS-MLPA panel. Bisulfite sequencing forward primers are indicated as F1/2 and reverse primers R1/2. Grey bar represents the MS-MLPA probes (LPO and RPO combined) and a black star stands for a CpG site targeted within GCGC restriction site. Figures are based on the UCSC Genome Browser (https://genome.ucsc.edu/cgi-bin/hgGateway). GH Reg Elems/GH Reg Elems (DE) stands for promoters, and vertical bars above the CpG site visualization indicate transcription starting sites.

Supplementary Table S1. MS-MLPA probes. Details of MS-MLPA probes of inflammation-associated genes custom panel.

Gene	Probe	Probe sequence	CpG position (GRCh38)	Total length (nt)
NTSR1	LPO	**GGGTTCCCTAAGGGTTGGA**CATTGGGGAACCGCGCGGTTTGGAGATCG	chr20:62708898	97
	RPO	GAGGCACCTGGAACCCGTGGCAAGCG**TCTAGATTGGATCTTGCTGGCAC**		
CD274	LPO	**GGGTTCCCTAAGGGTTGGA**TACCTGTTGGGGTTAATAAGAACAGGCAATGCA	chr9:5450802	104
	RPO	TCTGGCCTTCCTCCAGGCGCGATTCAGTT**TCTAGATTGGATCTTGCTGGCAC**		
PTGS2	LPO	**GGGTTCCCTAAGGGTTGGA**CATCTTGAGGGAAACCTGTGCGCCTGGGGCGGTGGAACTC	chr1:186680699	119
	RPO	GGGGAGGAGAGGGAGGGATCAGACAGGAGAGTT**TCATTCTAGATTGGATCTTGCTGGCAC**		
PPARG	LPO	**GGGTTCCCTAAGGGTTGGA**CATCTTGAGTCCATCTTGAGCACTCGGAGCCCGAGCCCGAGCC	chr3:12287965	124
	RPO	GCAGCCGCCGCCTGGGGCGCTTGGGTCGGTTCATGGTTC**TCTAGATTGGATCTTGCTGGCAC**		
PYCARD	LPO	**GGGTTCCCTAAGGGTTGGA**CGTGCTCCAATAAACCCAGTGAGAGCCAGCCCAGGTTTCCGGTCTGTA	chr16:31202988	134
	RPO	CCCGCTGGTGCAAGCCCAGAGACAAGCAGGCGCCACCCATGAGC**TCTAGATTGGATCTTGCTGGCAC**		
SOCS2	LPO	**GGGTTCCCTAAGGGTTGGA**CATCTTGAGTCCATCAGTGGGGGACCGCGGGGTCGGCGGAGGAGGCCATC	chr12:93572998	139
	RPO	GCAGCCGCCGCCTGGGGCGCTTGGGTCGGTTCATGGTTC**TCTAGATTGGATCTTGCTGGCAC**		
SOCS1	LPO	**GGGTTCCCTAAGGGTTGGA**CACACCTTCCTGGTGCGCGACAGCCGCCAGCGGAACTGCTTTTTCGCCCTTA	chr16:11255168	143
	RPO	GCGTGAAGATGGCCTCGGGACCCACGAGCATCCGCGTGCACTTTCAGTT**TCTAGATTGGATCTTGCTGGCAC**		

Note: Bold text indicates primer sequence, plain text stands for stuffer sequence (if needed), underlined text indicates hydridization sequence and red stands for a HhaI enzyme restriction site of which CpG site is assayed for the methylation. Total length is that of ligated LPO and RPO.
Abbreviations: LPO, left probe oligo; RPO, right probe oligo

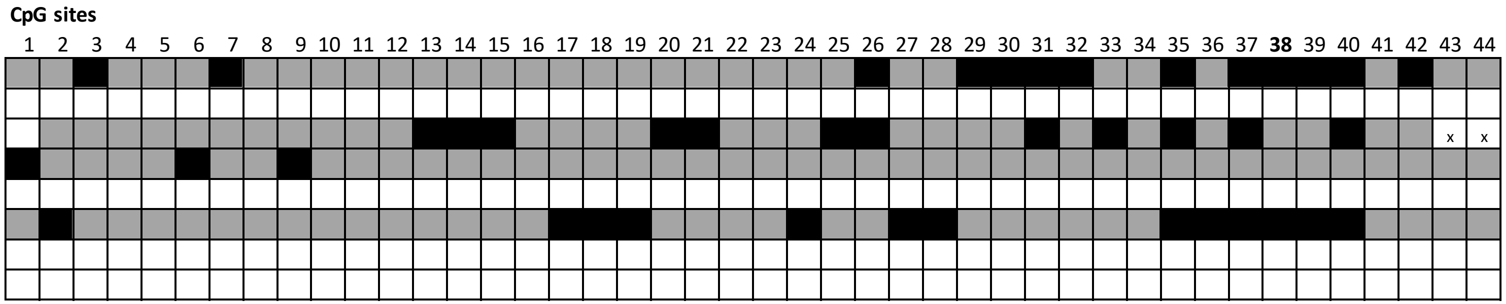

DNA sample	MS-MLPA	Cloning
HCA7	0.96	1.00
HCT116	0.04	0.00
LIM1215	0.75	0.79
RKO	0.43	0.30
SW480	0.02	0.00
T84	0.88	1.00
Healthy donor blood	0.03	0.00
Normal colon	0.21	0.08

Supplementary Figure S2. *NTSR1* cloning experiment compared to MS-MLPA. Sequencing and MS-MLPA results for investigated CpG islands of *NTSR1* in six colon cancer cell lines and two normal reference samples. Direct sequencing results are presented in the leftmost box, where each square refers to a CpG site (total n = 44) located in the amplified region. Black square indicates homozygous methylation (C/C), gray square heterozygous methylation (C/T), white square no methylation (T/T), and x stands for no result at the site in question in the corresponding cell line sample. CpG site no. 38 (in bold) is the site targeted by a custom-made MS-MLPA probe. MS-MLPA results (degree of methylation value, Dm) are presented in the rightmost box, as well as the proportion of methylated sequences obtained by sequencing after cloning.

Supplementary Table S2. Cut-off methylation dosage ratios (Dm) for hypermethylation, based on normal mucosae samples, for CIMP, inflammation-associated genes and MGMT probes.

CIMP		MLH1 I	MLH1 II	MLH1 III	MLH1 IV	IGF2 I	IGF2 II	IGF2 III	SOCS1 I	SOCS1 II	SOCS1 III	SOCS1 IV	NEUROG1 I	NEUROG1 II	NEUROG1 III	NEUROG1 IV	NEUROG1 V
CA-CRC normal (n = 12)	Average Dm	0,01	0,08	0,15	0,00	0,50	0,23	0,46	0,17	0,00	0,21	0,07	0,35	0,23	0,23	0,27	0,21
	Average + 2 SD	0,03	0,13	0,25	0,00	0,71	0,43	0,71	0,25	0,00	0,40	0,12	0,61	0,42	0,47	0,48	0,43
	Cut-off*	0,15	0,15	0,25	0,15	0,71	0,43	0,71	0,25	0,15	0,40	0,15	0,61	0,42	0,47	0,48	0,43
LS normal (n = 11/29)**	Average Dm	0,00	0,06	0,12	0,00	0,29	0,09	0,25	0,14	0,00	0,00	0,06	0,21	0,12	0,08	0,13	0,09
	Average + 2 SD	0,00	0,12	0,25	0,00	0,49	0,18	0,43	0,26	0,00	0,02	0,17	0,36	0,23	0,18	0,24	0,25
	Cut-off*	0,15	0,15	0,25	0,15	0,49	0,18	0,43	0,26	0,15	0,15	0,17	0,36	0,23	0,18	0,24	0,25

CA-CRC normal (n = 12)	Average Dm	NEUROG1 VI	CDKN2A I	CDKN2A II	CDKN2A III	CDKN2A IV	CRABP1 I	CRABP1 II	CRABP1 III	CRABP1 IV	CACNA1G I	CACNA1G II	CACNA1G III	RUNX3 I	RUNX3 II	RUNX3 III
	Average + 2 SD	0,27	0,11	0,07	0,16	0,11	0,20	0,26	0,21	0,14	0,16	0,11	0,09	0,15	0,13	0,14
	Cut-off*	0,49	0,17	0,12	0,21	0,22	0,43	0,44	0,40	0,22	0,28	0,19	0,13	0,26	0,21	0,23
		0,49	0,17	0,15	0,21	0,22	0,43	0,44	0,40	0,22	0,28	0,19	0,15	0,26	0,21	0,23
LS normal (n = 11/29)**	Average Dm															
	Average + 2 SD	0,14	0,09	0,05	0,13	0,02	0,09	0,19	0,13	0,08	0,15	0,07	0,03	0,11	0,08	0,09
	Cut-off*	0,30	0,18	0,11	0,24	0,08	0,16	0,35	0,27	0,15	0,31	0,13	0,09	0,21	0,18	0,20
		0,30	0,18	0,15	0,24	0,15	0,16	0,35	0,27	0,15	0,31	0,15	0,15	0,21	0,18	0,20

Inflammation-associated genes		NTSR1	CD274	PTGS2	PPARG	PYCARD	SOCS2	SOCS1
CA-CRC normal (n = 12)	Average Dm	0,29	0,17	0,18	0,16	0,24	0,12	0,17
	Average + 2 SD	0,37	0,27	0,27	0,26	0,39	0,18	0,28
	Cut-off*	0,37	0,27	0,27	0,26	0,39	0,18	0,28
LS normal (n = 11)	Average Dm	0,21	0,16	0,18	0,16	0,24	0,12	0,18
	Average + 2 SD	0,31	0,22	0,25	0,25	0,35	0,24	0,27
	Cut-off*	0,31	0,22	0,25	0,25	0,35	0,24	0,27

MGMT		124	140	160	172	190	215
CA-CRC normal (n = 17)	Average Dm	0,08	0,21	0,11	0,09	0,21	0,11
	Average + 2 SD	0,20	0,42	0,23	0,18	0,38	0,21
	Cut-off*	0,20	0,42	0,23	0,18	0,38	0,21
LS normal (n = 10)	Average Dm	0,03	0,13	0,08	0,08	0,16	0,09
	Average + 2 SD	0,11	0,22	0,13	0,14	0,25	0,15
	Cut-off*	0,15	0,22	0,15	0,15	0,25	0,15

* Cut off value was calculated as following: Average Dm + 2x standard deviation (SD) or technical threshold (Dm = 0.15), whichever was higher.
** Average Dm and SD values are based on LS samples used in this study (n = 11), cut-off values for hypermethylation are based on the previous study (n = 29) (refs. 19, 40).

Supplementary Table S3. Average methylation dosage ratios and their standard deviations (SD) of each CIMP markers in normal and tumorous samples of CA-CRC and LS patients. Statistically significant two-sided exact p values (Bonferroni corrected and raw p values) are given ($p < 0.05$), ns stands for non-significant p value.

	CA-CRC normal (n = 12)		CA-CRC tumors (n = 31)		LS normal (n = 11)		LS tumors (n = 29)	
**Probe**	**Average**	**SD**	**Average**	**SD**	**Average**	**SD**	**Average**	**SD**
**MLH1 I**	0,01	0,01	0,01	0,06	0,00	0,00	0,01	0,03
**MLH1 II**	0,08	0,03	0,09	0,06	0,06	0,03	0,07	0,05
**MLH1 III**	0,15	0,05	0,16	0,08	0,11	0,04	0,21	0,13
**MLH1 IV**	0,00	0,00	0,01	0,05	0,00	0,00	0,01	0,04
**IGF2 I**	0,50	0,11	0,61	0,19	0,33	0,07	0,57	0,18
**IGF2 II**	0,23	0,10	0,38	0,22	0,09	0,04	0,19	0,20
**IGF2 III**	0,46	0,12	0,68	0,16	0,25	0,09	0,56	0,24
**SOCS1 I**	0,17	0,04	0,17	0,06	0,14	0,04	0,15	0,07
**SOCS1 II**	0,00	0,00	0,03	0,10	0,00	0,00	0,08	0,17
**SOCS1 III**	0,21	0,10	0,25	0,12	0,00	0,00	0,00	0,00
**SOCS1 IV**	0,07	0,02	0,07	0,02	0,06	0,04	0,05	0,04
**NEUROG1 I**	0,35	0,13	0,50	0,20	0,20	0,05	0,40	0,22
**NEUROG1 II**	0,23	0,10	0,24	0,16	0,12	0,05	0,15	0,06
**NEUROG1 III**	0,23	0,12	0,38	0,23	0,07	0,06	0,20	0,21
**NEUROG1 IV**	0,27	0,11	0,42	0,24	0,13	0,06	0,31	0,20
**NEUROG1 V**	0,21	0,11	0,29	0,22	0,06	0,06	0,10	0,10
**NEUROG1 VI**	0,27	0,11	0,39	0,25	0,12	0,07	0,21	0,17
**CDKN2A I**	0,11	0,03	0,13	0,11	0,10	0,05	0,09	0,07
**CDKN2A II**	0,07	0,02	0,07	0,03	0,05	0,02	0,05	0,03
**CDKN2A III**	0,16	0,03	0,21	0,17	0,13	0,06	0,16	0,09
**CDKN2A IV**	0,11	0,06	0,12	0,16	0,01	0,02	0,08	0,12
**CRABP1 I**	0,20	0,12	0,32	0,22	0,09	0,04	0,19	0,20
**CRABP1 II**	0,26	0,09	0,35	0,18	0,18	0,07	0,27	0,17
**CRABP1 III**	0,21	0,10	0,25	0,12	0,14	0,06	0,18	0,11
**CRABP1 IV**	0,14	0,04	0,19	0,13	0,08	0,04	0,07	0,06
**CACNA1G I**	0,16	0,06	0,21	0,13	0,13	0,07	0,16	0,06
**CACNA1G II**	0,11	0,04	0,17	0,13	0,06	0,03	0,09	0,06
**CACNA1G III**	0,09	0,02	0,11	0,08	0,02	0,03	0,04	0,03
**RUNX3 I**	0,15	0,05	0,23	0,16	0,11	0,05	0,17	0,14
**RUNX3 II**	0,13	0,04	0,19	0,13	0,09	0,05	0,11	0,09
**RUNX3 III**	0,14	0,04	0,21	0,17	0,09	0,05	0,12	0,10

	CA-CRC normal vs. tumor		LS normal vs. tumor		CA-CRC normal vs. LS normal		CA-CRC tumors vs LS tumors	
**Probe**	**p value**	**raw p value**	**p value**	**raw p value**	**p value**	**raw p value**	**p value**	**raw p value**
**MLH1 I**	ns	ns	ns	ns	ns	ns	ns	ns
**MLH1 II**	ns	ns	ns	ns	ns	ns	ns	ns
**MLH1 III**	ns	ns	0,789	0,025	ns	ns	ns	ns
**MLH1 IV**	ns	ns	ns	ns	ns	ns	ns	ns
**IGF2 I**	ns	0,021	0,002	<0.0001	0,012	<0.001	ns	ns
**IGF2 II**	ns	0,024	ns	ns	0,002	<0.0001	0,005	<0.001
**IGF2 III**	0,010	<0.001	0,003	<0.0001	0,001	<0.0001	ns	ns
**SOCS1 I**	ns	ns	ns	ns	ns	ns	ns	ns
**SOCS1 II**	ns	ns	ns	ns	ns	ns	ns	ns
**SOCS1 III**	ns	ns	ns	ns	<0.0001	<0.0001	<0.0001	<0.0001
**SOCS1 IV**	ns	ns	ns	ns	ns	ns	ns	0,027
**NEUROG1 I**	ns	0,026	0,129	0,004	0,040	0,001	ns	0,042
**NEUROG1 II**	ns	ns	ns	ns	ns	0,016	ns	0,019
**NEUROG1 III**	ns	ns	ns	ns	0,039	0,001	ns	0,004
**NEUROG1 IV**	ns	0,040	0,217	0,007	0,017	<0.001	ns	ns
**NEUROG1 V**	ns	ns	ns	ns	0,009	<0.001	<0.001	<0.0001
**NEUROG1 VI**	ns	ns	ns	ns	ns	0,003	ns	0,004
**CDKN2A I**	ns	ns	ns	ns	ns	ns	ns	0,033
**CDKN2A II**	ns	ns	ns	ns	ns	ns	0,029	<0.001
**CDKN2A III**	ns	ns	ns	ns	ns	ns	ns	ns
**CDKN2A IV**	ns	0,032	ns	ns	<0.0001	<0.0001	ns	0,027
**CRABP1 I**	ns	ns	ns	ns	0,030	0,001	ns	0,006
**CRABP1 II**	ns	ns	ns	ns	ns	ns	ns	ns
**CRABP1 III**	ns	ns	ns	ns	ns	ns	ns	0,035
**CRABP1 IV**	ns	ns	ns	ns	ns	0,002	<0.001	<0.0001
**CACNA1G I**	ns	ns	ns	ns	ns	ns	ns	ns
**CACNA1G II**	ns	ns	ns	ns	ns	0,004	ns	0,002
**CACNA1G III**	ns	ns	ns	ns	<0.001	<0.0001	<0.001	<0.0001
**RUNX3 I**	ns	ns	ns	ns	ns	ns	ns	ns
**RUNX3 II**	ns	ns	ns	ns	ns	0,043	0,019	<0.001
**RUNX3 III**	ns	ns	ns	ns	ns	0,050	ns	0,008

Supplementary Table S4. Average methylation dosage ratios (Dm) and standard deviations (SD) of each MGMT probe in normal and tumorous samples of CA-CRC and LS patients. Statistically significant two-sided exact p values (Bonferroni corrected and raw p values) are given ($p < 0.05$), ns stands for non-significant p value.

	CA-CRC normal (n = 17)		CA-CRC tumors (n = 30)		LS normal (n = 10)		LS tumors (n = 16)	
**Probe**	**Average**	**SD**	**Average**	**SD**	**Average**	**SD**	**Average**	**SD**
**MGMT 124**	0,08	0,06	0,12	0,11	0,03	0,04	0,08	0,12
**MGMT 140**	0,21	0,10	0,30	0,17	0,13	0,05	0,19	0,14
**MGMT 160**	0,11	0,06	0,13	0,09	0,08	0,02	0,12	0,13
**MGMT 172**	0,09	0,04	0,10	0,08	0,08	0,03	0,10	0,11
**MGMT 190**	0,21	0,09	0,23	0,10	0,16	0,05	0,23	0,12
**MGMT 215**	0,11	0,05	0,14	0,10	0,09	0,03	0,13	0,11

	CA-CRC normal vs. tumor		LS normal vs. tumor		CA-CRC normal vs. LS normal		CA-CRC tumors vs. LS tumors	
	**p value**	**raw p value**	**p value**	**raw p value**	**p value**	**raw p value**	**p value**	**raw p value**
**MGMT 124**	ns	ns	ns	ns	ns	ns	ns	ns
**MGMT 140**	ns	ns	ns	ns	ns	0,020	ns	0,035
**MGMT 160**	ns	ns	ns	ns	ns	ns	ns	ns
**MGMT 172**	ns	ns	ns	ns	ns	ns	ns	ns
**MGMT 190**	ns	ns	ns	ns	ns	ns	ns	ns
**MGMT 215**	ns	ns	ns	ns	ns	ns	ns	ns

Supplementary Table S5. Methylation dosage ratios (Dm) of inflammation-associated genes in cancer cell lines and their corresponding normal reference samples (highlighted with light green). Dm values of cell lines exceeding those of the tissue-specific reference samples (highlighted with light green), or the technical threshold of 0.15, are highlighted in brown and considered hypermethylated. Cancer cell lines in bold text indicate those used in bisulphite sequencing and to optimize the custom-made MS-MLPA panel of inflammatory genes.

Cell line	Tissue	Morphology	MSI status	CIMP status	*NTSR1*	*CD274*	*PTGS2*	*PPARG*	*PYCARD*	*SOCS2*	*SOCS1*
BT-549	breast, ductal carcinoma	epithelial	MSS	neg	0,14	0,24	0,05	0,04	1,13	0,74	0,07
CAL-51	breast, mammary gland, basal cell adenocarcinoma	epithelial-like	MSI	neg	0,15	0,43	0,09	0,05	1,10	0,58	0,67
ZR-75-1	breast, mammary gland, ductal carcinoma	epithelial	MSS	pos	0,52	0,65	0,97	0,00	0,34	0,22	0,04
MCF12A	breast, mammary gland, normal	epithelial	MSS		0,17	0,15	0,15	0,15	0,15	1,04	0,49
CACO-2	colon, adenocarcinoma	epithelial	MSS	pos	0,49	0,26	0,12	0,09	1,13	0,00	0,24
**HCA7**	colon, adenocarcinoma	epithelial	MSI	pos	0,96	0,06	0,02	0,04	0,11	0,88	0,95
**HCT116**	colon, carcinoma	epithelial	MSI	pos	0,04	0,37	0,51	0,02	1,14	0,88	0,53
HCT15	colon, adenocarcinoma	epithelial	MSI	pos	1,08	0,12	0,21	0,04	1,03	0,88	0,64
KM12	colon, adenocarcinoma	epithelial	MSI	pos	0,89	0,23	0,06	0,02	1,04	0,85	0,93
**LIM1215**	colon, carcinoma	epithelial	MSI	pos	0,75	0,12	0,02	0,05	1,16	0,24	0,57
LoVo	colon, adenocarcinoma	epithelial	MSI	pos	1,07	0,06	0,09	0,09	1,03	0,71	0,50
**RKO**	colon, carcinoma	epithelial	MSI	pos	0,43	0,03	0,55	0,73	1,17	1,04	0,07
**SW480**	colon, adenocarcinoma	epithelial	MSS	pos	0,02	0,15	0,05	0,05	1,14	1,07	0,06
SW837	rectum, adenocarcinoma	epithelial	MSS	neg	1,03	0,04	0,03	0,00	0,55	0,00	0,53
**T84**	colon, carcinoma (derived from lung metastasis)	epithelial	MSS	pos	0,88	0,11	0,98	0,06	0,84	0,10	0,08
CCD-112CoN	colon, normal	fibroblast		neg	0,12	0,08	0,02	0,05	0,21	0,38	0,11
Normal colon (Dr.P set; Amsbio)	colon, normal	epithelial			0,21	0,12	0,21	0,13	0,33	0,15	0,36
AN3CA	endometrium, adenocarcinoma	epithelial	MSI	pos	0,18	0,19	1,02	0,38	1,16	0,99	0,98
ECC-1	edometrium, adenocarcinoma	epithelial	MSI	pos	0,23	0,93	0,03	0,01	1,05	1,10	1,02
Normal endometrium (Dr.P set; Amsbio)	endometrium, normal	epithelial			0,15	0,15	0,15	0,15	0,29	0,15	0,15
CAOV3	ovario, serous adenocarcinoma	epithelial	MSS	neg	0,16	0,05	0,05	0,07	0,93	0,00	0,10
ES2	ovario, clear cell adenocarcinoma	epithelial	MSS	pos	0,25	0,04	0,07	0,03	0,78	0,87	0,79
SKOV3	ovario, serous adenocarcinoma	epithelial	MSI	pos	0,26	0,91	0,10	0,01	1,11	1,04	0,94
Normal ovario*	ovario, normal fallopian tube	epithelial			0,15	0,20	0,15	0,15	0,37	0,15	0,15
TKM	healthy control	blood			0,03	0,13	0,03	0,01	0,07	0,14	0,04

* Cut off value was calculated as following: Average Dm + 2x standard deviation (SD) of normal ovario (n = 10) or technical threshold (Dm = 0.15), whichever was higher.

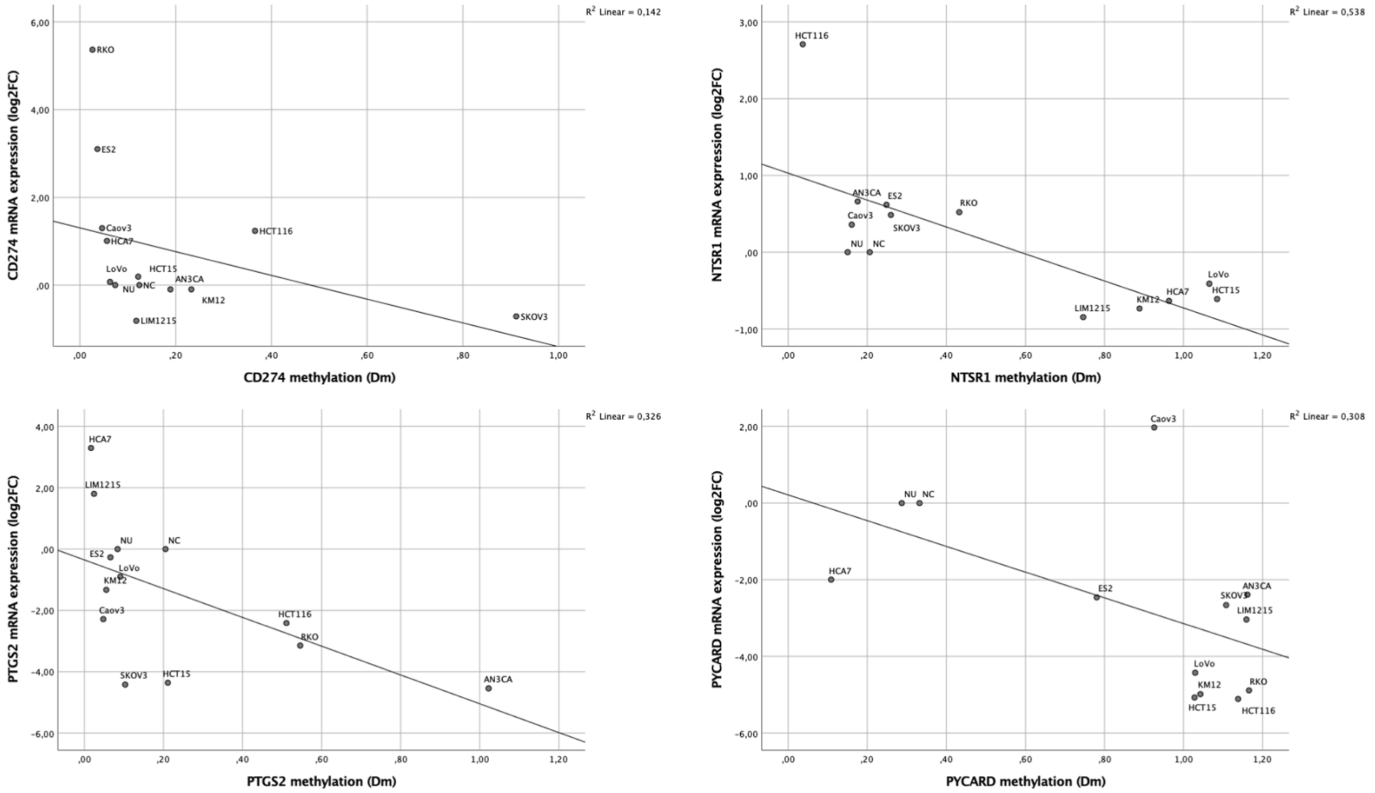

Supplementary Figure S3. Correlation between expression and methylation of inflammation-associated genes in cell lines. Significant inverse correlation in *CD274* (r = -0.680, p = 0.010), *NTSR1* (r = -0.680, p = 0.010), *PTGS2* (r = -0.735, p = 0.004) and *PYCARD* (r = -0.564, p = 0.045). Cell lines' characteristics and inflammation-associated gene methylation are presented in Supplementary Table S5. NC, normal colon; NU, normal uterine (endometrium).

Supplementary Table S6. Bisulfite sequencing primers used to investigate methylation status of 12 selected genes. Gene names in bold refer to those included in the final custom-made MS-MLPA panel.

Gene	Forward primer	Reverse primer	Product size	Reference (PMID)	T_a used in PCR
NTSR1	F1: TTTTTTTYGTYGTTATTTTTTATTGTTGGG	R1: CCTAACTCCAAAACCCRTAATTCCTC	344	26334593	57
CD274	F1: TTGGATTTGTTGTTTTGGGTAGAGG	R1: TAACTCTACTACCCCCTAAACCATC	297	This study	57
CD274	F2: ATGGTTTAGGGGGTAGTAGAGTTA	R2: CACAAATCATATTACTAACAAACAACT	256	This study	52
PTGS2	F1: AGATTTTTGGAGAGGAAGTTAAGTGT	R1: AAAAAATAATCCCCACTCTCCTATC	202	27722841	57
PTGS2	F2: GATTTGTAGTGAGYGTTAGGAGTA	R2: CCAAATACTCACCTATATAACTAAAC	186	Modified from 10945606	52
PPARG	F1: GTGTGAAGGGTAAGTTATTTTGGT	R1: AACTCCRAATACRCTAAACCCC	423	This study	56
PPARG	F2: TTTGGTYGGGGGTATTTTTTTAAATTT	R2: AACACCCRTACTCTAACCTAC	463	This study	56
PYCARD	F1: GATTTTAYGATATGTGGGAGAGGATT	R1: AACCTCTAAATTAAAACCCCAAACC	272	This study	55
SOCS2	F2: GGGATTYGTATTGATTTTAAGGAAGG	R2: ATCRACTCCCTACCTATCTAACC	243	This study	52
SOCS1	F1: TGTAGGATGGTAGTATATAATTAGGTGGT	R1: TAATACTCCAACAACTCRAAAAAACAATC	467	15386345	57
CFS2	F1: GTATGTGAATGTTATTTAGGAGGTT	R1: AACACTATCTCAACAAAACTTAATCT	549	This study	56
CXCL12	F2: GAGGGGYGAAGGGGATGGGT	R2: ACCCAAAACCTCRCCAAAACCTCC	353	This study	66
FOXP3	F2: CTAGYGGGGGTTTTATAGGGGT	R2: CTCCATTAACTATTCTCTCTCTCTA	524	This study	52
PDCD1	F2: GAGATTYGGGAGTGGTTTTTTGTTT	R2: TCTCCTTATATTCTATACTAAACATTTT	298	This study	52
SOCS3	F1: GAGTAGTGATTAAATATTATAAGAAGG	R1: TCCCTCRAACTTCCCCTAAAC	286	This study	52
SOCS3	F2: TGYGTTTTAAGATTTTTAGTTTTAAGAG	R2: TTCTTAATCCCAAACTAAATCTTAAC	214	This study	52

Note: Reference given as a PubMed ID. T_a is the annealing temperature (°C) in the first cycle of the PCR program.
